# Agomelatine Alleviates Depressive-like Behaviors by Suppressing Hippocampal Oxidative Stress in the Chronic Social Defeat Stress Model

**DOI:** 10.3390/antiox14040410

**Published:** 2025-03-28

**Authors:** Yan Zhu, Ye Li, Zhaoying Yu, Xiao Chen, Tian Lan, Meijian Wang, Shuyan Yu

**Affiliations:** 1 Department of Physiology, School of Basic Medical Sciences, Cheeloo College of Medicine, Shandong University, Jinan 250012, China; 202100220017@mail.sdu.edu.cn (Y.Z.); 202390000063@sdu.edu.cn (Y.L.); 202100411012@mail.sdu.edu.cn (Z.Y.); 202120737@mail.sdu.edu.cn (X.C.); 202220836@mail.sdu.edu.cn (T.L.); 2 Department of Endocrinology, Qilu Hospital, Shandong University, Qingdao, 758 Hefei Road, Qingdao 266035, China; wangmeijian@sdu.edu.cn; 3 Shandong Key Laboratory of Mental Disorders and Intelligent Control, School of Basic Medical Sciences, Cheeloo College of Medicine, Shandong University, Jinan 250012, China

**Keywords:** oxidative stress, mitochondrial dysfunction, synaptic dysfunction, agomelatine, depression

## Abstract

Major depressive disorder (MDD) is a common psychiatric disorder characterized by significant mood disturbances and cognitive impairments. Chronic stress, particularly social defeat stress, plays a crucial role in the etiology of depression, with oxidative stress being a pivotal factor in its pathophysiology. Consequently, identifying effective strategies to mitigate oxidative stress and prevent the progression of depression is of paramount importance. Agomelatine, an atypical antidepressant with melatonergic and serotonergic properties, has shown promise in treating MDD due to its unique mechanisms of action. In this study, we aimed to investigate whether agomelatine could ameliorate behavioral deficits in a chronic social defeat stress (CSDS) mouse model. CSDS mice were administered agomelatine (50 mg/kg, intraperitoneally) and exhibited significant reductions in both anxiety-like and depressive-like behaviors in behavioral tests. Further analysis revealed that agomelatine treatment effectively reduced oxidative damage in the hippocampus of CSDS mice. Additionally, agomelatine attenuated mitochondrial dysfunction and restored synaptic plasticity, as evidenced by an increased density of excitatory synapses and enhanced neuronal activity. These findings suggest that agomelatine may exert therapeutic effects by reducing oxidative stress, preserving mitochondrial function, and enhancing synaptic plasticity, providing new insights into its potential as a treatment for chronic social defeat stress-induced depression.

## 1. Introduction

Major depressive disorder (MDD) is a widespread and heterogeneous psychiatric condition that impacts over 300 million individuals globally, constituting one of the leading causes of disability [1,2]. It is characterized by a wide range of symptoms, including anhedonia, cognitive impairment, and mood disturbances [3,4,5]. Extensive research indicates that multiple interrelated mechanisms contribute to MDD, including hypothalamic–pituitary–adrenal (HPA) axis dysfunction, monoamine neurotransmitter imbalance, immune–inflammatory activation, genetic and epigenetic anomalies, structural and functional brain remodeling, oxidative stress, and social psychological stress [6]. However, no single hypothesis can fully account for the pathophysiology of MDD, as these mechanisms are intricately linked and mutually influence one another [7,8]. Among various risk factors, chronic psychosocial stress plays a significant role in the development of depression [9,10], inducing long-lasting neurobiological alterations in mood-regulating brain regions, such as the hippocampus. Studies have reported structural abnormalities and impaired neurogenesis in the hippocampus of individuals with MDD [11]. The chronic social defeat stress (CSDS) model is extensively utilized to replicate depressive-like behaviors resulting from social stress in experimental settings [12,13,14]. However, despite substantial advances in understanding the pathophysiology of MDD, the precise molecular mechanisms linking social defeat stress to depression remain poorly understood, thereby limiting the development of more effective therapeutic strategies.

Oxidative stress has emerged as a critical factor in the pathophysiology of MDD, as evidenced by numerous studies [15,16]. Elevated levels of reactive oxygen species (ROS) and diminished antioxidant defenses, including decreased glutathione (GSH) and superoxide dismutase (SOD) activity, are commonly observed in individuals with MDD, both in the peripheral and central systems [17,18,19,20]. These oxidative stress imbalances cause damage to cellular macromolecules, such as lipids, proteins, and nucleic acids, leading to neuronal dysfunction and structural changes, which are particularly pronounced in the hippocampus and prefrontal cortex [21,22,23]. Furthermore, numerous studies have demonstrated that markers of oxidative damage, including lipid peroxidation products and oxidative modifications to DNA/RNA, are consistently elevated in major depressive disorder (MDD) and are closely correlated with disease severity, chronicity, and treatment resistance [24,25,26,27,28]. Notably, oxidative stress has been implicated in reduced neurogenesis and hippocampal atrophy, both hallmark features of depression-related brain deficits [29,30]. Moreover, antidepressant treatments have shown promise in restoring the balance between oxidative and antioxidative systems, highlighting the potential of oxidative stress not only as a biomarker but also as a therapeutic target in MDD [31,32].

Agomelatine, a melatonergic and serotonergic agent, has demonstrated potent antidepressant effects through its dual mechanism of action. It serves as an MT1/MT2 melatonin receptor agonist and a 5-HT_2C_ serotonin receptor antagonist [33,34]. Melatonin plays a pivotal role in depression by regulating circadian rhythms, modulating neurotransmitter systems, and exerting neuroprotective and anti-inflammatory effects [8], while serotonin (5-HT) is an essential neuromodulator involved in mood regulation, neuroplasticity, and stress responses [6]. This dual mechanism facilitates improvements in sleep quality by promoting slow-wave sleep and synchronizing disrupted circadian rhythms while enhancing hippocampal neurogenesis and increasing brain-derived neurotrophic factor (BDNF) levels [35,36]. Preclinical models and clinical trials provide evidence that agomelatine effectively alleviates depressive symptoms, mitigates circadian disruptions, and reverses mood-related neuronal impairments [37,38,39]. Additionally, it exhibits a favorable safety profile with lower risks of withdrawal symptoms and weight gain than selective serotonin reuptake inhibitors (SSRIs) and serotonin–norepinephrine reuptake inhibitors (SNRIs) [40]. These attributes position agomelatine as a valuable treatment option for major depressive disorder (MDD) [41]. However, there is limited research investigating the relationship between agomelatine and oxidative stress in the chronic social defeat stress (CSDS) model. Further studies are warranted to explore its potential role in mitigating oxidative damage in neuropsychiatric conditions.

In this study, we sought to elucidate the potential mechanisms responsible for the neuroprotective effects of agomelatine on chronic social defeat stress (CSDS)-induced depressive behaviors. Our results revealed that agomelatine markedly alleviated depressive and anxiety-like behaviors through the inhibition of oxidative stress-induced damage, mitigation of mitochondrial dysfunction, and enhancement of synaptic plasticity. These findings offer novel insights into the therapeutic potential of agomelatine, particularly in addressing the oxidative stress and synaptic dysfunction associated with depressive disorders related to social defeat stress.

## 2. Materials and Methods

### 2.1. Animals and Experimental Design

Male C57BL/6J mice (6–8 weeks) and retired male CD-1 mice (16–20 weeks) were procured from Charles River Laboratories. The animals were housed in cages under controlled conditions, including a 12 h light/dark cycle (lights on from 07:00 to 19:00), 55 ± 5% humidity, and a temperature of 22 ± 2 °C, with unrestricted access to food and water. All experiments were conducted in accordance with the international guidelines for animal research set by the Council of International Medical Organizations. Agomelatine was sourced from Aladdin (Shanghai, China) and was dissolved in 1% hydroxyethyl cellulose (MedChemExpress, Monmouth Junction, NJ, USA) at a concentration of 50 mg/kg. The dosage and intraperitoneal injection schedule for agomelatine administration were determined based on prior studies [40,42,43].

**Figure 1:**
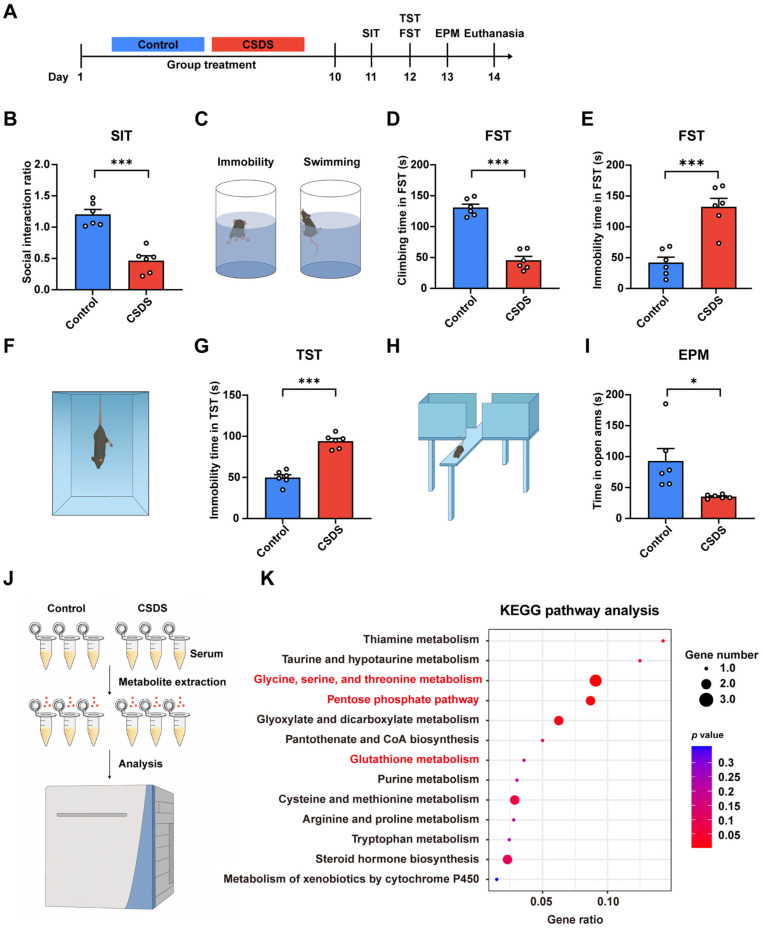
Behavioral deficits and metabolomic alterations in CSDS mice. (**A**) Schematic representation of the CSDS procedure and subsequent behavioral tests conducted in mice. (**B**) Social interaction ratio in the SIT (*n * =  6 per group). (**C**–**E**) Climbing time and immobility time in the FST (*n*  =  6 per group). (**F**,**G**) Immobility time in the TST (*N*  =  6 per group). (**H**,**I**) Time spent in open arms in the EPM (*n*  =  6 per group). (**J**,**K**) Bar plot from KEGG enrichment analysis based on significant differential metabolites in the serum (*n* =  3 per group). Data are presented as the means ± SEMs. * *p* < 0.05, and *** *p* < 0.001 compared to Control, analyzed using a two-tailed, unpaired Student’s *t*-test. For detailed statistical data, see in Supplementary Table S1.

The study was conducted using two experiments. In the first experiment, C57BL/6J mice were randomly divided into the following two groups (*N* = 15 per group): Control, Chronic Social Defeat Stress (CSDS). The experimental timeline is shown in Figure 1A. In the second experiment, mice were randomly allocated to the following four groups (*N* = 18 per group): Control, CSDS, CSDS + Vehicle, and CSDS + Agomelatine [50 mg/kg, intraperitoneally (i.p.)]. During the 10-day CSDS modeling period, mice received intraperitoneal injections of agomelatine or vehicle 60 min prior to the social defeat procedures. This treatment regimen was maintained for 10 consecutive days, and then behavioral assessments and tissue collection were conducted.

### 2.2. Chronic Social Defeat Stress (CSDS) Model

The CSDS paradigm was conducted as previously described [44,45]. Aggressors were selected from retired male CD-1 mice according to the following criteria: a latency of less than 60 s to initiate an attack on C57BL/6J mice, and an interval of less than 3 min between subsequent attacks. Over 10 consecutive days, experimental mice were introduced into the home cage of a different CD-1 mouse each day, where they endured a 5 min physical attack daily. Following the attack, the intruder mice were separated from the aggressor by a mesh partition and subjected to continuous social stress from the CD-1 mouse for 24 h until the next attack. Each C57BL/6J mouse encountered a different resident CD-1 mouse daily to prevent habituation to a single aggressor. Behavioral assessments were conducted 24 h after the final attack.

### 2.3. Behavioral Tests

The behavioral tests were conducted following previously established protocols with some minor modifications [45,46,47].

#### 2.3.1. Social Interaction Test (SIT)

The Social Interaction Test (SIT) was conducted in two phases. In the initial phase, experimental mice were exposed to an open-field arena (40 cm × 40 cm × 40 cm) containing an empty wire-mesh cage (10 cm × 7 cm × 18 cm) for 2.5 min. In the second phase, the same mice were reintroduced to the arena for another 2.5 min, this time with a novel and aggressive CD-1 mouse confined within the wire-mesh cage. The time spent by the experimental mice in the interaction zone around the wire-mesh cage was recorded and analyzed using TopScan software 3.00 (CleverSys Inc., Reston, VA, USA). The social interaction (SI) ratio was calculated as [time spent in the interaction zone with the CD-1 mouse/time spent in the interaction zone without the CD-1 mouse]. Mice with an SI ratio less than 1 were classified as susceptible, while those with an SI ratio greater than 1 were classified as resilient. All mice used in the experiment were categorized as susceptible. To prevent cross-contamination between tests, both the open field arena and the wire-mesh cage were thoroughly cleaned with 75% alcohol after each trial. In the first experiment, the SIT was performed on 12 mice (*N* = 6 per group: Control, CSDS). In the second experiment, 32 mice (*n* = 8 per group: Control, CSDS, CSDS + Vehicle, CSDS + AGO) were assessed.

#### 2.3.2. Three-Chambered Social Interaction Test (TBSIT)

The apparatus for the three-chambered social interaction test comprised a rectangular box partitioned into three equally sized chambers (20 cm × 40 cm × 21 cm). The test protocol consisted of three distinct stages. In the initial stage, experimental mice were introduced to the central chamber and allowed a 10-min habituation period to acclimate to the environment. In the second stage, referred to as the sociability test, an unfamiliar C57BL/6J male mouse (“Stranger 1”), with no prior interaction with the experimental mice, was confined in a wire-mesh cage (10 cm × 7 cm × 18 cm) placed in either the left or right chamber. An identical empty wire-mesh cage was positioned in the opposite chamber. The experimental mice were then introduced into the middle chamber and permitted to explore all three chambers for 10 min. The duration spent by the experimental mice near each cage (Stranger 1 or empty) was recorded. In the third stage, designated as the social preference test, a novel C57BL/6J male mouse (“Stranger 2”) was placed in an identical wire-mesh cage in the chamber opposite to that containing Stranger 1. The experimental mice were again given 10 min to explore the apparatus freely. The time spent investigating each cage (Stranger 1 or Stranger 2) was recorded. For the sociability test, the preference index was determined as [time spent around the cage with Stranger 1/time spent around the empty cage]. For the social preference test, the preference index was calculated as [time spent around the cage with Stranger 2/time spent around the cage with Stranger 1]. The TBSIT was conducted on 32 mice (*N* = 8 per group: Control, CSDS, CSDS + Vehicle, CSDS + AGO).

#### 2.3.3. Open Field Test (OFT)

The open field test was utilized to assess anxiety-related behaviors and locomotor activities of mice within a 40 cm × 40 cm × 40 cm open-field arena. Each experimental mouse was positioned at the center of the arena and permitted to explore freely for 5 min. The total duration spent in the central zone (20 cm × 20 cm) during the 5-min session was quantified using TopScan software. Following each trial, the arena was sanitized with 75% ethanol solution. OFT was performed on 32 mice (*N* = 8 per group: Control, CSDS, CSDS + Vehicle, CSDS + AGO).

#### 2.3.4. Elevated Plus Maze (EPM)

The elevated plus maze (EPM) test was utilized to assess anxiety-related behaviors in experimental animals. The apparatus comprised two enclosed arms (30 cm × 6 cm × 15 cm) and two open arms (30 cm × 6 cm). Each mouse was individually positioned on the central platform, which was oriented towards one of the open arms. The mice were permitted to explore the EPM apparatus for a duration of 5 min, during which the time spent in both the enclosed and open arms was recorded using TopScan software. Following each trial, the cross maze was sanitized with 75% ethanol to maintain a standardized testing environment. In the first experiment, the EPM was conducted on 12 mice (*N* = 6 per group: Control, CSDS). In the second experiment, 32 mice (*n* = 8 per group: Control, CSDS, CSDS + Vehicle, CSDS + AGO) were evaluated.

#### 2.3.5. Tail Suspension Test (TST)

The tail suspension test was utilized to evaluate behavioral despair in mice. Mice were suspended by their tails using a binder clip positioned 1 cm from the tail tip and 20 cm above the ground. Each trial lasted for 6 min, with immobility time being measured during the final 4 min. Following each trial, the apparatus was sanitized with 75% ethanol to maintain a sterile environment for subsequent trials. In the first experiment, the TST was performed on 12 mice (*N* = 6 per group: Control, CSDS). In the second experiment, 32 mice (*N* = 8 per group: Control, CSDS, CSDS + Vehicle, CSDS + AGO) were assessed.

#### 2.3.6. Forced Swim Test (FST)

The forced swim test (FST) was employed to assess depressive-like behaviors in mice. Each experimental mouse was individually placed in a transparent glass cylinder (12 cm in diameter, 25 cm in height) filled with 10 cm of water maintained at 25 ± 2 °C. The test duration was 6 min, with immobility time recorded during the final 4 min. Following each trial, the water was completely replaced to prevent any residual odors from influencing subsequent test results. In the first experiment, the FST was performed on 12 mice (*N* = 6 per group: Control, CSDS). In the second experiment, 32 mice (*N* = 8 per group: Control, CSDS, CSDS + Vehicle, CSDS + AGO) were assessed.

#### 2.3.7. Sucrose Preference Test (SPT)

The sucrose preference test (SPT) was employed to assess anhedonia symptoms. The experiment spanned four consecutive days, during which mice were individually housed for testing. On the first day, the animals were habituated to two identical bottles containing 1% sucrose solution for a period of 24 h. On the second day, one bottle contained 1% sucrose solution, while the other contained water, both of which were available for 24 h. On the third day, the mice were subjected to 24 h of water and food deprivation. On the fourth day, the mice were given unrestricted access to two bottles, with one containing 1% sucrose solution and the other containing water. After 12 h, the bottle positions were swapped to control for side bias. At the end of 24 h, the bottles were weighed, and sucrose preference was calculated as [1% sucrose consumption/(sucrose consumption + water consumption)] × 100%. The SPT was conducted on 32 mice (*N* = 8 per group: Control, CSDS, CSDS + Vehicle, CSDS + AGO).

### 2.4. Measurement of MDA, SOD, GSH, and LDH

The activities of the antioxidant enzymes in hippocampus tissues were measured using specific assay kits—the malondialdehyde (MDA) content assay kit (BC0025), superoxide dismutase (SOD) activity assay kit (BC5165), reduced glutathione (GSH) content assay kit (BC1175), and L-lactate dehydrogenase (L-LDH) activity assay kit (BC0685), all purchased from Solarbio (Beijing, China). Before conducting the assays, hippocampus tissues were pre-treated as follows. For the MDA, SOD, and LDH assays, the tissues were dissociated by a tissue lyser, followed by centrifuging the homogenate at 8000 rpm for 10 min at 4 °C. The supernatant was subsequently collected for analysis. Protein concentrations of the supernatant were quantified using a BCA assay kit (Cwbio, Taizhou, China). For the GSH assay, tissues were first weighed and treated with reagent 1 from the GSH kit. After thorough mixing, the extracts were centrifuged at 12,000× *g* for 10 min at 4 °C, and the supernatant was collected for analysis. After these pre-treatment steps, the assays were performed following the manufacturer’s guidelines. In the first experiment, MDA and SOD activities were measured in 12 mice (*N* = 6 per group: Control, CSDS). In the second experiment, MDA, SOD, GSH, and LDH activities were assessed in 24 mice (*N* = 6 per group: Control, CSDS, CSDS + Vehicle, CSDS + AGO).

### 2.5. Western Blotting (WB)

Hippocampal tissues were homogenized using RIPA lysis buffer containing protease and phosphatase inhibitors. Following centrifugation at 12,000 rpm for 20 min at 4 °C, the supernatants containing isolated proteins were collected. Protein concentrations were determined using a bicinchoninic acid (BCA) assay kit after mixing the extracts with 2× loading buffer and heating at 100 °C for 8 min. Proteins were then separated by SDS-PAGE on 12.5% gels and transferred onto PVDF membranes (Millipore, Burlington, MA, USA). The membranes were blocked with 5% BSA for 1 h, followed by overnight incubation at 4 °C with the following primary antibodies: Rabbit anti-Nrf2 (1:2000, Proteintech, Chicago, IL, USA, 16396-1-AP), Rabbit anti-HO-1 (1:1000, Abcam, Cambridge, UK, ab13243), Rabbit anti-Cytc (1:1000, Affinity, Changzhou, china AF0146), and Mouse anti-GAPDH (1:50,000, Proteintech, Chicago, IL, USA, 60004-1-Ig). On the following day, the membranes were incubated with secondary antibodies—anti-rabbit HRP-conjugated (1:2000, ZSGB-Bio, Beijing, China, ZB-2301) or anti-mouse HRP-conjugated (1:2000, ZSGB-Bio, Beijing, China, ZB-2305)—for 1 h at room temperature. Protein bands were identified using the Enhanced Chemiluminescence Kit (Vazyme, Nanjing, China), and signals were visualized with the Chemiluminescence Imager (Tanon-4600). Images were subsequently analyzed using ImageJ software 1.54F (National Institutes of Health, NIH, Bethesda, MD, USA). In the first experiment, Western blot analysis was performed on 12 mice (*N* = 6 per group: Control, CSDS) to assess Nrf2 and HO-1 expression. In the second experiment, Western blot analysis was conducted on 24 mice (*N* = 6 per group: Control, CSDS, CSDS + Vehicle, CSDS + AGO) to evaluate Nrf2, HO-1, and Cytc expression.

### 2.6. DHE Staining

To assess reactive oxygen species (ROS) production in tissue samples, 30 μm cryosections were incubated with 10 μM dihydroethidium (DHE; Sigma, Louis, MO, USA) for 30 min at 37 °C. Subsequently, the sections were incubated with DAPI for 10 min. Fluorescence images were captured using a HOOKE S3000 spinning-disk confocal microscope (HOOKE Instruments Ltd., Changchun, China) and analyzed quantitatively using ImageJ software. The fluorescence intensity values were normalized to those of the control group. DHE staining was conducted on 12 mice in the first experiment (*N* = 6 per group: Control, CSDS) and on 24 mice in the second experiment (*N* = 6 per group: Control, CSDS, CSDS + Vehicle, CSDS + AGO).

### 2.7. Immunofluorescence

Immunofluorescence staining was conducted on frozen coronal brain sections from mice. Following post-fixation and graded dehydration, brain tissues were sectioned into 30 μm thick sections using a cryostat microtome. The slices were rinsed three times with PBS for 5 min each, followed by blocking in a fluorescence-blocking solution supplemented with 0.2% Triton X-100, 2.5% bovine serum albumin (BSA), and 5% donkey serum for 1 h at room temperature. Subsequently, the slices were incubated with primary antibodies overnight at 4 °C. The next day, sections were incubated with secondary antibodies for 1 h at room temperature. The primary antibodies used were Mouse anti-DNA/RNA Damage antibody (1:500, Abcam, ab62623), Rabbit anti-4-hydroxynonenal (1:500, Abmart, Shanghai, China, PC6313), Mouse anti-PSD-95 (1:100, Santa Cruz Biotechnology, Santa Cruz, CA, USA, sc-32290), and Rabbit anti-VGLUT1 (1:100, Bioworld, Bloomington, MN, USA, bs75067). Secondary antibodies included Alexa Fluor 488 Goat anti-Mouse IgG H&L (1:500, Abcam, Cambridge, UK, ab150113),Goat anti-Rabbit FITC (1:200, Abcam, Cambridge, UK, ab97050), and Alexa Fluor 568 Donkey anti-Rabbit IgG H&L (1:500, Invitrogen, Carlsbad, CA, USA, A10042). Sections were then counterstained with DAPI for 10 min. Fluorescence images were acquired using a spinning-disk confocal microscope (HOOKE S3000, HOOKE Instruments Ltd., Changchun, China). For analysis, one representative image per animal from six animals was selected, and fluorescence intensity was quantified using ImageJ software. Results were presented as ratios relative to those of the control group. In the first experiment, immunofluorescence staining was performed on 12 mice (*N* = 6 per group: Control, CSDS) to assess the level of 8-OHdG. In the second experiment, immunofluorescence was conducted on 24 mice (*N* = 6 per group: Control, CSDS, CSDS + Vehicle, CSDS + AGO) to evaluate 8-OHdG and 4-HNE, as well as the colocalization of VGLUT1 and PSD95.

### 2.8. Transmission Electron Microscopy (TEM)

Transmission electron microscopy (TEM) was utilized to examine the ultrastructure of hippocampal neurons. Small samples of hippocampal tissue (1 mm^3^) were meticulously dissected and fixed in 2.5% glutaraldehyde at 4 °C for 2–3 h. The tissues were then rinsed five times with PBS (0.1 M, pH 7.4) and subjected to 1% osmium tetroxide fixation for 1.5 h. Subsequently, the tissues underwent graded ethanol dehydration followed by infiltration with propylene oxide overnight. After resin embedding, ultrathin sections (65 nm) were prepared using a Leica UC7 ultramicrotome and stained with 2% uranyl acetate for 25 min, followed by citrate for 7 min. Mitochondrial morphology was observed and imaged using a Hitachi HT-7800 transmission electron microscope. TEM was conducted on 24 mice (*N* = 6 per group: Control, CSDS, CSDS + Vehicle, CSDS + AGO).

### 2.9. Hippocampal Slice Preparation and Electrophysiological Recordings

Mice were anesthetized using 1% sodium pentobarbital and subsequently decapitated. Brain slices (300 μm) were obtained using a vibratome (VT1200s, Leica, Wetzlar, Germany) in ice-cold artificial cerebrospinal fluid (ACSF) containing 125 mM NaCl, 2.5 mM KCl, 25 mM NaHCO_3_, 1.25 mM NaH_2_PO_4_, 1 mM MgCl_2_, 2 mM CaCl_2_, and 25 mM glucose, with 1 mM pyruvate added. The brain slices were then immediately transferred to ASCF at 32 °C for at least 30 min at 33 °C, followed by room-temperature storage until further use. All solutions were continuously bubbled with 95% O2/5% CO2. Whole-cell recordings were performed using glass pipettes (4 to 6 MΩ) filled with an internal solution containing 105 mM potassium gluconate, 30 mM KCl, 4 mM Mg-ATP, 0.3 mM Na-GTP, 0.3 mM EGTA, 10 mM HEPES, and 10 mM sodium phosphocreatine, with the pH set to 7.25–7.30. For mEPSC recordings, neurons were held and recorded at a specific potential in ASCF using a MultiClamp 700B amplifier. Data were analyzed using the Mini Analysis Program Ver 6.0 (Synaptosoft). Electrophysiological recordings were performed on 24 mice (*N* = 6 per group: Control, CSDS, CSDS + Vehicle, CSDS + AGO).

### 2.10. LC–MS/MS Analysis

LC–MS/MS analysis was conducted as previously described [48,49,50,51,52,53]. Serum samples were preconditioned by mixing with methanol, followed by centrifugation at 12,000 rpm for 10 min at 4 °C and filtration through a 0.22 μm membrane. The supernatants (2 μL injection volume) were separated using a Vanquish UHPLC System (Thermo Fisher Scientific, Waltham, MA, USA) equipped with an ACQUITY UPLC^®^ HSS T3 column. Separation was performed by gradient elution at a flow rate of 0.3 mL/min and column temperature of 40 °C, with mobile phases consisting of 0.1% formic acid in acetonitrile and 0.1% formic acid in water (positive ESI mode) or acetonitrile with 5 mM ammonium formate (negative ESI mode). Mass spectrometry was conducted on an Orbitrap Exploris 120 mass spectrometer (Thermo Fisher Scientific, USA) with an ESI ion source, which was operated in both positive and negative modes under simultaneous MS1 and data-dependent MS/MS (Full MS-ddMS2) acquisition. Raw data were converted to mzXML format using MSConvert in ProteoWizard software package (v3.0.8789) and subsequently processed with the R XCMS package (v3.12.0). Metabolites were identified based on accurate mass and MS/MS spectra by matching against databases including HMDB, MassBank, KEGG, LipidMaps, mzCloud, and a custom database developed by Panomix Biomedical Tech Co., Ltd. (Suzhou, China). Multivariate statistical analysis was conducted using the R ropls package (v1.22.0), and metabolites with a *p* value < 0.05 and a VIP value > 1 were considered differential metabolites. Pathway enrichment analysis was then performed with MetaboAnalyst 6.0. LC–MS/MS analysis was conducted on 6 mice (*N* = 3 per group: Control, CSDS).

### 2.11. Statistical Analysis

All data were expressed as the means ± SEMs and analyzed using GraphPad Prism software (version 10.1.2). Statistical significance was set at *p* < 0.05. Data normality was assessed individually, and non-parametric tests were utilized when the data deviated from normality (*p* < 0.05). For comparisons between two groups, homogeneity of variance was assessed via the F-test. If *p* > 0.05, indicating equal variances, an unpaired t-test was conducted. In cases where variances were unequal (*p* < 0.05), Welch’s *t*-test was employed. For comparisons involving four groups, the Brown–Forsythe test was performed to assess homogeneity of variance. In the presence of heterogeneous variances (*p* < 0.05), one-way analysis of variance (ANOVA) followed by Dunnett’s T3 post hoc test was performed. When variances were homogeneous, multiple comparisons were performed using one-way ANOVA followed by Bonferroni post hoc test. Statistical methods were not used to predetermine sample sizes, and the chosen sample sizes were consistent with those reported in previous studies [12,54]. Additionally, a post hoc power analysis was performed using G*Power to confirm that the sample sizes in key experiments provided sufficient statistical power (≥0.8) to detect meaningful effects.

## 3. Results

### 3.1. CSDS Modeling Induced Behavioral Disorders and Oxidative Stress

The chronic social defeat stress (CSDS) model was employed to induce depressive-like behaviors in mice (Figure 1A). After 10 day of exposure to CSDS, significant social avoidance behaviors were observed. The social interaction test (SIT) revealed a markedly reduced social interaction (SI) ratio in CSDS-exposed mice compared to the control group (Control), indicating social dysfunction in the CSDS group (Figure 1B). In the forced swim test (FST), CSDS-exposed mice exhibited prolonged immobility times and diminished climbing behavior (Figure 1C–E). Additionally, the tail suspension test (TST) demonstrated prolonged immobility times in the CSDS group, further supporting the presence of behavioral despair (Figure 1F,G). The elevated plus maze (EPM) test indicated a reduction in the time spent in the open arms by CSDS-exposed mice, suggesting heightened anxiety levels (Figure 1H,I). Collectively, these findings demonstrate that chronic social defeat stress leads to the manifestation of social avoidance, depressive, and anxiety-related behaviors in mice.

To elucidate the underlying mechanisms of CSDS-induced depression, we conducted LC-MS/MS-based non-targeted metabolomics analysis on serum samples from Control and CSDS-exposed mice. This analysis identified 117 differential metabolites, including gluconic acid, L-Cysteine, glyoxylic acid, and D-Ribose 5-phosphate. KEGG pathway enrichment analysis of significantly altered metabolites indicated that CSDS exposure was closely associated with pathways related to oxidative stress, including glycine, serine, and threonine metabolism, the pentose phosphate pathway (PPP), and glutathione metabolism (Figure 1J,K). Collectively, these findings suggest that CSDS may induce an imbalance in oxidative stress, potentially contributing to depressive and anxiety-like behaviors in mice.

### 3.2. Oxidative Stress Was Observed in the Hippocampi of the CSDS-Induced Depression Group

**Figure 2:**
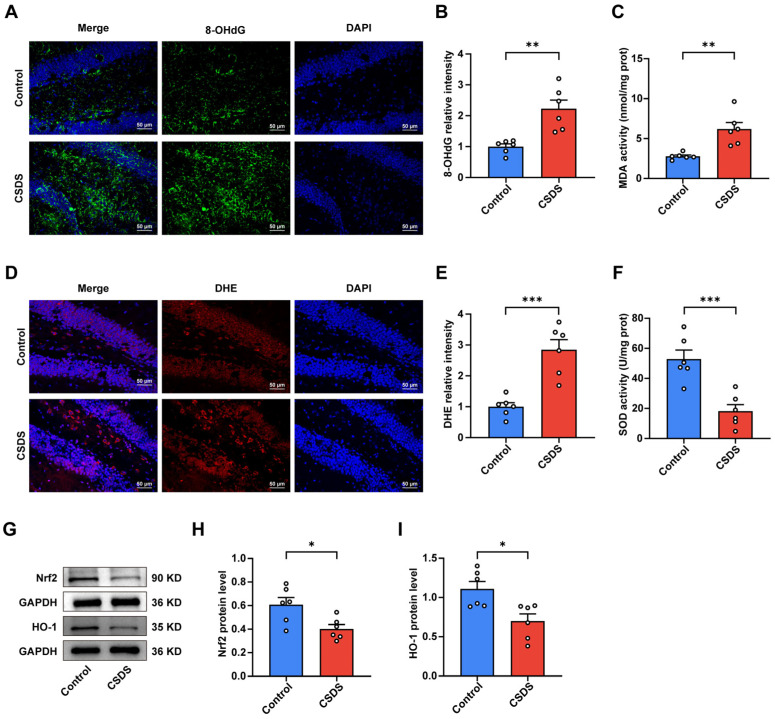
Overactivated oxidative stress in the hippocampus after CSDS exposure. (**A**,**B**) Representative images of 8-OHdG staining (green) and quantification of relative fluorescent intensity (*n*  =  6 per group). Nuclei (blue) are stained with DAPI. Scale bar is 50 μm. (**C**) Activity of MDA in the hippocampus (*n*  =  6 per group). (**D**,**E**) Representative images of DHE staining (red) and quantification of relative fluorescent intensity (*n*  =  6 per group). Nuclei (blue) are stained with DAPI. Scale bar is 50 μm. (**F**) Activity of SOD in the hippocampus (*n*  =  6 per group). (**G**–**I**) The protein expression of Nrf2 (**H**), HO-1 (**I**) (*n*  =  6 per group). Parallel gels were used to analyze the expression levels of HO-1. Data are expressed as the means  ±  SEMs. * *p* < 0.05, ** *p* < 0.01, and *** *p* < 0.001 compared to Control, analyzed using a two-tailed, unpaired Student’s *t*-test. For detailed statistical data, see in Supplementary Table S1.

To validate these findings, we quantified the expression levels of oxidative stress-related markers in hippocampal tissues. Consistent with the metabolomic data, CSDS-exposed mice exhibited a significant increase in 8-hydroxy-2′-deoxyguanosine (8-OHdG), a marker of DNA oxidative damage, and malondialdehyde (MDA), an indicator of lipid peroxidation (Figure 2A–C). Additionally, reactive oxygen species (ROS) levels, as measured by dihydroethidium (DHE) fluorescence, were markedly elevated, while superoxide dismutase (SOD) activity, a critical antioxidant enzyme, was significantly diminished in CSDS-exposed mice, indicating an imbalance between oxidative and antioxidative components (Figure 2D–F). Furthermore, exposure to CSDS resulted in a significant downregulation of nuclear factor erythroid 2-related factor 2 (Nrf2) and heme oxygenase-1 (HO-1) expression in the hippocampus (Figure 2G–I). Collectively, these findings suggest that oxidative stress may be a pivotal contributor to the development of CSDS-induced depressive-like behaviors.

### 3.3. Agomelatine Reverses Social Disorders After CSDS Exposure

**Figure 3:**
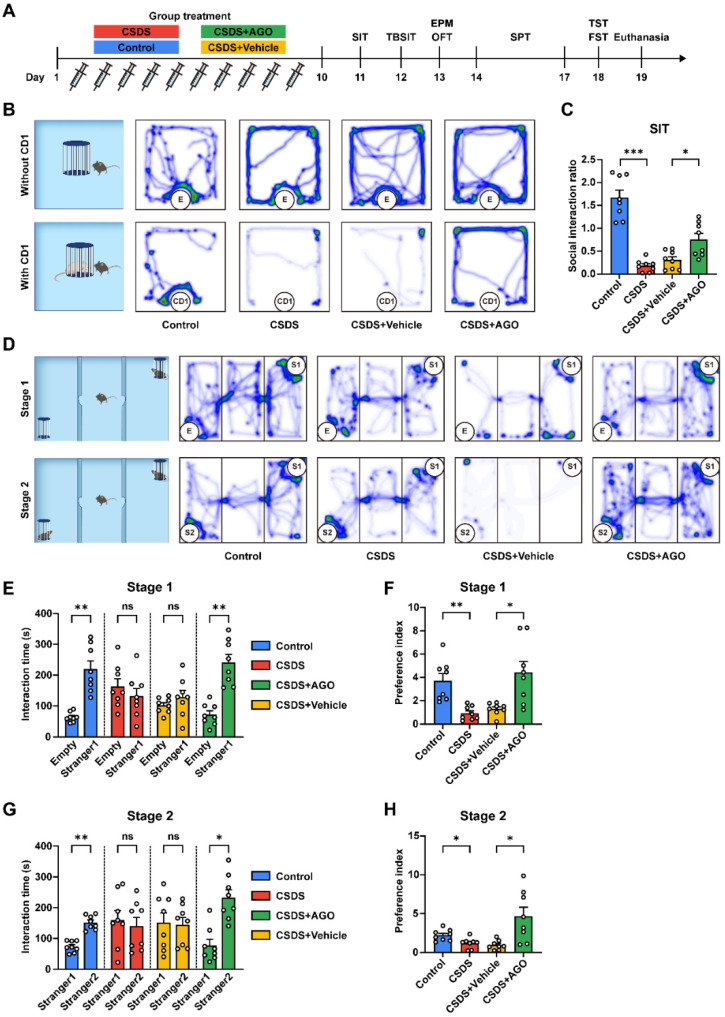
Effects of agomelatine treatment on social avoidance in CSDS-exposed mice. (**A**) Schematic representation of the CSDS procedure, agomelatine treatment, and subsequent behavioral tests in mice. (**B**,**C**) Heat maps of path tracing and the social interaction ratio in the SIT (*n*  =  8 per group). (**D**–**H**) Heat maps of path tracing in the TBSIT (**D**), interaction time (**E**,**G**), and the preference index (**F**,**H**) in the first and second stages (*n*  =  8 per group). Data are presented as the means  ±  SEMs. ns *p*  >  0.05, * *p*  <  0.05, ** *p*  <  0.01, *** *p*  <  0.001. Comparisons were performed between the control and CSDS groups or CSDS + Vehicle and CSDS + AGO groups using Student’s *t*-tests (**E**,**G**) or one-way ANOVA followed by Dunnett’s T3 post hoc test (**C**,**F**,**H**). For detailed statistical data, see in Supplementary Table S1.

To examine the potential role of agomelatine in social behavior, CSDS mice were subjected to intraperitoneal injection of agomelatine. Behavioral evaluations related to social behaviors, including the social interaction test (SIT) and the three-chambered social interaction test, were conducted after modeling. Consistent with prior findings, CSDS-exposed mice with vehicle injection showed notable disorders in social behavior, while agomelatine treatment ameliorated these deficits, reversing the observed social disorders. As illustrated in Figure 3B,C, CSDS exposure induced a significant decrease in the social interaction (SI) ratio. Nonetheless, administration of agomelatine significantly increased the SI ratio compared to that in the CSDS + Vehicle group. In the three-chambered sociability test, which evaluates the preference for a chamber containing a conspecific mouse versus an empty chamber, CSDS-exposed mice showed a markedly diminished interest in the chamber containing the mouse. Agomelatine treatment, however, significantly improved the preference index compared to that in the CSDS + Vehicle group (Figure 3D–F). Additionally, in the three-chamber social preference test, designed to assess discrimination between a novel mouse and a familiar mouse, CSDS-exposed mice displayed a decreased interest in socializing with the novel mice compared to those in the control group, while agomelatine treatment effectively restored the preference index, as demonstrated by increased interaction with the novel mouse compared to the familiar one (Figure 3D,G,H). These findings indicate that CSDS exposure led to the deficits in social interaction. Importantly, treatment with agomelatine improved the social behavior of CSDS-exposed mice.

### 3.4. Agomelatine Ameliorates Depressive and Anxiety-like Behaviors Induced by CSDS Exposure

**Figure 4:**
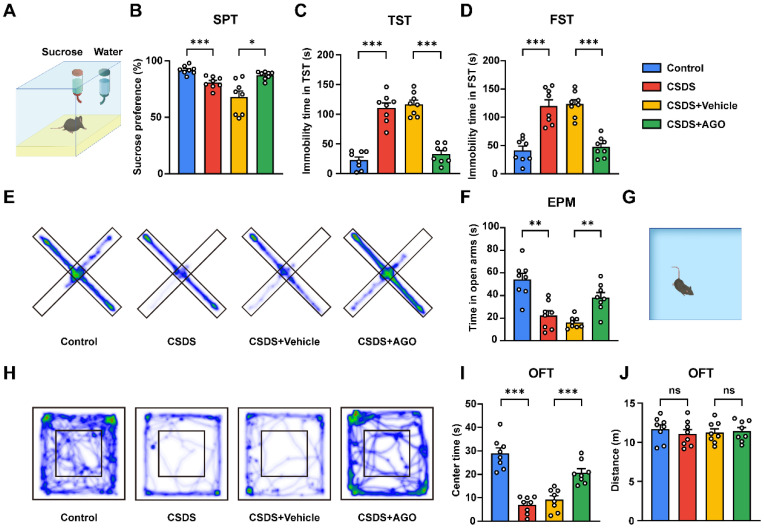
Effects of agomelatine treatment on depressive and anxiety-like behaviors in CSDS mice. (**A**,**B**) Sucrose preference in SPT (*n*  =  8 per group). (**C**) Immobility time in TST (*n*  =  8 per group). (**D**) Immobility time in FST (*n*  =  8 per group). (**E**,**F**) Heat maps of path tracing and time spent in open arms in the EPM (*n*  =  8 per group). (**G**–**J**) Heat maps of path tracing (**H**), time spent exploring the center zone (**I**) and the total distance (**J**) in the OFT (*n*  =  8 per group). Data are expressed as means  ±  SEMs. ns *p*  >  0.05, * *p*  <  0.05, ** *p*  <  0.01, *** *p*  <  0.001. Statistical comparisons were performed between Control and CSDS or CSDS + Vehicle and CSDS + Ago groups using one-way ANOVA followed by Dunnett’s T3 post hoc test (**B**–**D**,**F**,**I**) or one-way ANOVA followed by Bonferroni post hoc test (**J**). For detailed statistical data, see in Supplementary Table S1.

To validate the efficacy of the CSDS-induced depression model in eliciting behavioral markers of depression and to evaluate the therapeutic potential of agomelatine, we conducted a series of behavioral tests associated with depression and anxiety. These tests comprised the sucrose preference test (SPT), tail suspension test (TST), forced swimming test (FST), elevated plus maze (EPM), and open field test (OFT) (Figure 3A). In the SPT, agomelatine treatment significantly attenuated the reduction in sucrose consumption observed in CSDS-exposed mice, thereby restoring sucrose preference (Figure 4A,B). The TST and FST revealed a marked decrease in immobility time in the CSDS + AGO group compared to the CSDS + Vehicle group (Figure 4C,D). Anxiety-like behaviors were assessed using the OFT and EPM. Mice in the CSDS + AGO group exhibited reduced anxiety-like behavior, as evidenced by increased time spent in the center zone of the OFT and the open arms of the EPM, compared to those in the CSDS + Vehicle group (Figure 4E–I). Collectively, these findings provide compelling evidence that agomelatine administration effectively reversed depressive and anxiety-like behaviors, underscoring its therapeutic potential for alleviating CSDS-induced depression and anxiety.

### 3.5. Agomelatine Suppressed Oxidative Stress in the Hippocampal of Mice CSDS Model

**Figure 5:**
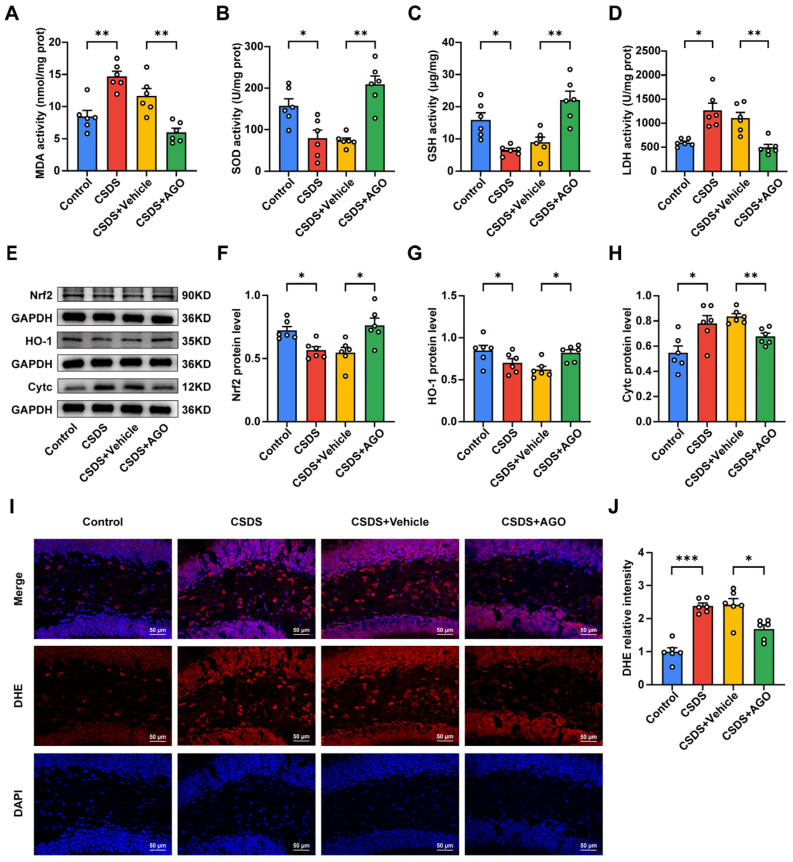
Agomelatine treatment suppressed oxidative stress in hippocampus of CSDS-exposed mice. (**A**–**D**) Activities of MDA (**A**), SOD (**B**), GSH (**C**), and LDH (**D**) in the hippocampus (*n*  =  6 per group). (**E**–**H**) The protein expression of Nrf2 (**F**), HO-1 (**G**), and Cytc (**H**) in the hippocampus (*n*  =  6 per group). Parallel gels were used to analyze the expression levels of HO-1. (**I**,**J**) Representative images of DHE staining (red) and quantification of relative fluorescent intensity (*n*  =  6 per group). Nuclei are stained with DAPI (blue). Scale bar is 50 μm. Data are presented as the means  ±  SEMs. * *p*  <  0.05, ** *p*  <  0.01, *** *p*  <  0.001. Statistical analyses were performed using one-way ANOVA followed by Dunnett’s T3 post hoc test for comparisons between the control and CSDS groups or CSDS + Vehicle and CSDS + AGO groups. For detailed statistical data, see in Supplementary Table S1.

**Figure 6:**
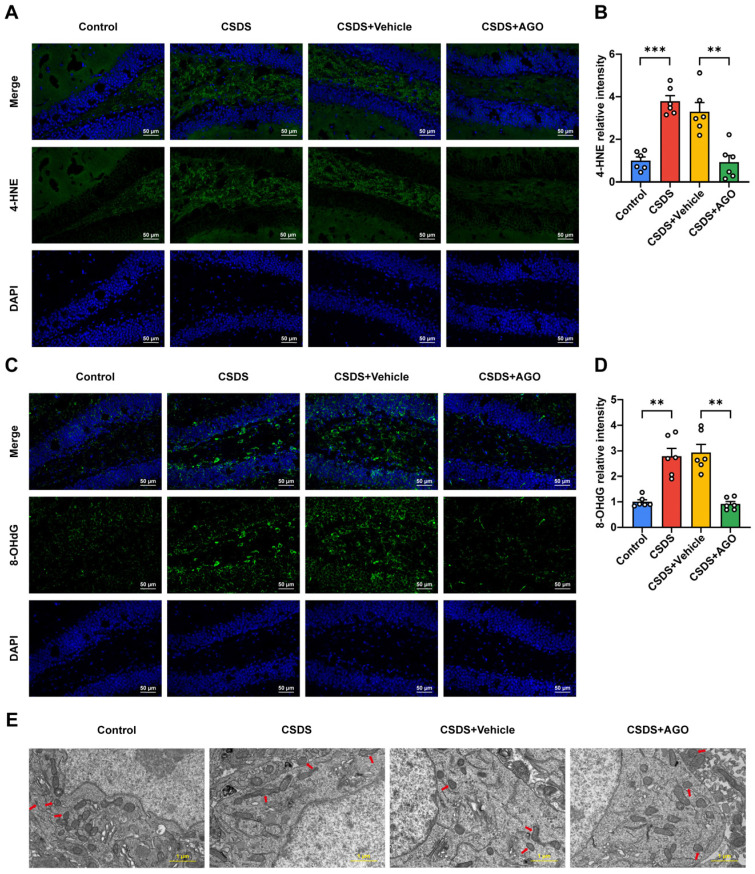
Agomelatine improved oxidative damage and mitochondrial morphology in the hippocampi of CSDS-exposed mice. (**A**,**B**) Representative images of 4-HNE staining (green) and quantification of relative fluorescent intensity (*n*  =  6 per group). Nuclei (blue) were counterstained with DAPI (blue). Scale bar is 50 μm. (**C**,**D**) Representative images of 8-OHdG staining (green) and quantification of relative fluorescent intensity (*n*  =  6 per group). Nuclei were counterstained with DAPI (blue). Scale bar is 50 μm. (**E**) Representative electron micrographs of hippocampal neurons (*n*  =  6 per group). Scale bar is 1 μm. Arrows indicate mitochondria. Data are presented as the means ± SEMs. ** *p* < 0.01, *** *p* < 0.001. Statistical comparisons were performed using one-way ANOVA followed by Dunnett’s T3 post hoc test between the control and CSDS groups or CSDS + Vehicle and CSDS + AGO groups. For detailed statistical data, see in Supplementary Table S1.

Considering the critical contribution of oxidative stress in the pathogenesis and progression of depression, we evaluated the impact of agomelatine on oxidative stress by assessing the activities of malondialdehyde (MDA), superoxide dismutase (SOD), glutathione (GSH), and lactate dehydrogenase (LDH) in the hippocampus. As illustrated in Figure 5A–D, chronic social defeat stress (CSDS) exposure significantly decreased SOD and GSH activities while markedly increasing MDA and LDH levels compared to the control group. Conversely, agomelatine treatment effectively reversed these changes (Figure 5A–D). Consistent with these observations, dihydroethidium (DHE) staining demonstrated a significant increase in reactive oxygen species (ROS) levels in the hippocampus of CSDS-exposed mice relative to controls. Agomelatine administration significantly mitigated this ROS elevation (Figure 5I,J). Considering the essential role of the Nrf2/HO-1 pathway in regulating antioxidant gene expression, we investigated its involvement in the potential effects of agomelatine on this pathway. Western blot analysis showed that CSDS exposure notably decreased the expression of both Nrf2 and HO-1 in the hippocampus, whereas agomelatine treatment substantially restored their levels (Figure 5E–G). Since oxidative stress can lead to damage in lipids, proteins, and DNA, we also examined markers of oxidative damage. In the hippocampus of CSDS-exposed mice, we observed a significant increase in 4-hydroxynonenal (4-HNE), a marker of lipid peroxidation, which was significantly reduced following agomelatine treatment (Figure 6A,B). Additionally, the expression of 8-hydroxy-2′-deoxyguanosine (8-OHdG) was significantly elevated in the hippocampus of CSDS-exposed mice but was notably decreased upon agomelatine administration (Figure 6C,D). Collectively, these findings indicate that oxidative stress in the hippocampus is a critical consequence of chronic social defeat stress (CSDS) exposure. Agomelatine demonstrates protective effects by modulating oxidative stress and alleviating subsequent oxidative damage, thereby offering valuable insights into its potential therapeutic mechanisms.

### 3.6. Agomelatine Ameliorates Mitochondrial Damage After CSDS Exposure

Considering the potential role of mitochondrial dysfunction in exacerbating oxidative stress in major depressive disorder (MDD), we conducted a detailed examination of mitochondrial integrity in the hippocampus using transmission electron microscopy (TEM). Chronic social defeat stress (CSDS) exposure led to significant structural alterations in mitochondria, characterized by swelling and cristae loss in neuronal cells. However, treatment with agomelatine effectively alleviated these pathological changes (Figure 6E). Mitochondrial damage, marked by increased outer membrane permeabilization, triggers the release of cytochrome C (Cytc) into the cytoplasm. To further elucidate this mechanism, we evaluated Cytc expression levels via Western blot analysis. As illustrated in Figure 5E,H, CSDS exposure significantly elevated Cytc levels in the hippocampus, whereas agomelatine treatment reversed these effects. Collectively, these findings suggest that agomelatine may mitigate CSDS-induced mitochondrial damage.

### 3.7. Agomelatine Ameliorates Synaptic Plasticity Impairment in the Hippocampi of CSDS-Exposed Mice

**Figure 7:**
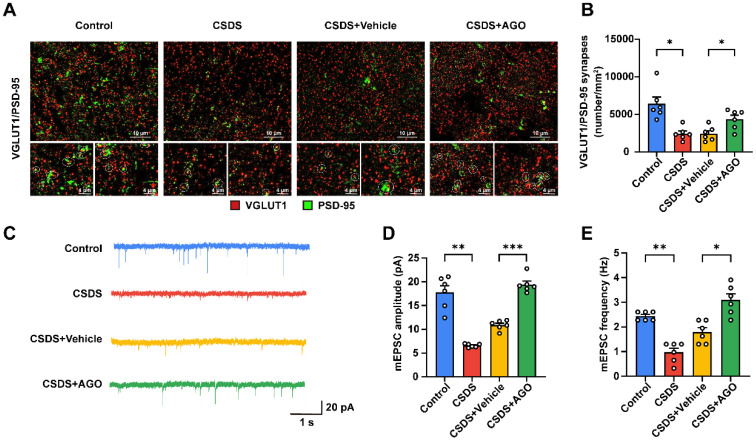
Agomelatine treatment improved synaptic injury in the hippocampi of CSDS-exposed mice. (**A**,**B**) Representative images of dual immunofluorescence staining of VGLUT1 (red) and PSD-95 (green) and quantification of the number of colocalized VGLUT1 and PSD-95 puncta (*n*  =  6 per group). (**C**–**E**) Representative raw traces (**C**), amplitude (**D**), and frequency (**E**) of mEPSCs (*n*  = 6 from 3 cells per group). Data are expressed as the means ± SEMs. * *p* < 0.05, ** *p* < 0.01, *** *p* < 0.001. Statistical comparisons of quantification of VGLUT1 and PSD-95 colocalization as well as mEPSC amplitude were conducted using one-way ANOVA followed by Dunnett’s T3 post hoc test (**B**,**D**) or a non-parametric test (**E**) between the control and CSDS groups or CSDS + Vehicle and CSDS + Ago groups. For detailed statistical data, see in Supplementary Table S1.

Considering the high vulnerability of synapses to oxidative stress, we evaluated synaptic function in the hippocampus through double immunofluorescent staining for vesicular glutamate transporter 1 (VGLUT1) and postsynaptic density protein-95 (PSD-95). Our results demonstrated a significant reduction in VGLUT1/PSD-95 colocalization in hippocampal neurons from chronic social defeat stress (CSDS)-exposed mice, indicating a substantial decrease in excitatory synapse density. However, treatment with agomelatine effectively restored this synaptic density (Figure 7A,B). To further investigate the functional implications of these synaptic changes, we performed whole-cell patch-clamp recordings. Consistent with our expectations, CSDS exposure resulted in marked reductions in both the frequency and amplitude of miniature excitatory postsynaptic currents (mEPSCs). Notably, agomelatine treatment not only reversed these effects but also significantly enhanced both the frequency and amplitude of mEPSCs (Figure 7C–E). Collectively, these findings suggest that CSDS exposure disrupts both structural and functional synaptic plasticity in the hippocampus, an effect that was mitigated by agomelatine.

## 4. Discussion

MDD is a prevalent psychiatric condition that affects approximately 4.4% of the global population [11]. It is characterized by persistent low mood, anhedonia, and heightened vulnerability to stress, imposing a significant burden on both individuals and public health systems [9,12,55]. Chronic psychosocial stress is a critical risk factor for MDD, influencing its onset and progression through intricate interactions between genetic and environmental factors [9]. Additionally, oxidative stress has emerged as a pivotal contributor to the pathophysiology of MDD [20], with evidence indicating that oxidative damage can lead to neuronal dysfunction and impair neuroplasticity [21]. Despite the availability of various antidepressant therapies, such as selective serotonin reuptake inhibitors (SSRIs), treatment efficacy remains suboptimal [56]. Many patients experience delayed therapeutic effects, low remission rates, and substantial nonresponse, with up to 30% of individuals failing to benefit from current medications [57,58]. Additionally, SSRIs and serotonin–norepinephrine reuptake inhibitors (SNRIs) are frequently associated with adverse effects such as gastrointestinal disturbances, sexual dysfunction, and withdrawal symptoms upon discontinuation [59,60]. This has spurred interest in alternative treatments, such as agomelatine, which, beyond its action on melatonergic systems, may offer additional benefits by modulating key biological pathways that influence mood and stress responses [34,61]. Notably, agomelatine demonstrates a more favorable tolerability profile but requires careful monitoring for hepatic toxicity [60,62]. These challenges highlight the necessity for further investigation into the molecular mechanisms underlying stress-induced susceptibility to MDD. A deeper understanding of these mechanisms is crucial for identifying novel therapeutic targets and enhancing treatment outcomes for individuals with MDD.

In this study, we investigated the antidepressant and anxiolytic effects of agomelatine, along with its potential underlying mechanisms. Our findings demonstrated that agomelatine significantly alleviated depressive and anxiety-like behaviors induced by CSDS. The observed therapeutic effects were associated with the modulation of oxidative stress within the hippocampus. Furthermore, agomelatine exhibited neuroprotective properties by preventing CSDS-induced mitochondrial damage and mitigating synaptic dysfunction in the hippocampal region. These results highlight the promising therapeutic potential of agomelatine for treating depression and anxiety disorders while also offering new insights into the role of oxidative stress in these conditions.

Recent studies have shown that MDD is closely linked to both structural and functional impairments in the hippocampus [63], a brain region critical for mood and cognitive regulation [64]. In particular, the hippocampus is severely affected during the onset of depression [65]. The CSDS model, which simulates stressful life events, has proven effective and become widely used for studying depression [66]. Emerging evidence suggests that CSDS induces HPA axis hyperactivation, impaired neurogenesis, neuroinflammation, and oxidative stress, leading to hippocampal dysfunction and depressive-like behaviors [45,67,68]. Therefore, in this study, we focused on the hippocampi of CSDS-exposed mice to explore the molecular mechanisms associated with depression.

To elucidate the potential mechanisms underlying depression, we performed metabolomic sequencing analysis on the hippocampal region of both CSDS and control mice. The results demonstrated significant alterations in plasma metabolites, and subsequent KEGG pathway analysis indicated that CSDS-induced depression disrupts multiple metabolic pathways, with oxidative stress emerging as a pivotal factor. Extensive evidence supports the notion that oxidative stress contributes to hippocampal damage induced by stress and is a key factor in the onset and progression of depression [69]. Furthermore, given the well-documented antioxidant properties of agomelatine across various systems, including cardiovascular, hepatic, and renal systems [33,70,71], as well as its efficacy in other depression models such as chronic restraint stress (CRS) and chronic mild stress (CMS) [72,73], we aimed to investigate its potential therapeutic effects in the CSDS model.

Oxidative stress, arising from the impairment of the antioxidant defense system [74,75], disrupts the equilibrium between pro-oxidant and antioxidant components within cells [76], including glutathione (GSH), superoxide dismutase (SOD), lactate dehydrogenase (LDH), and reactive oxygen species (ROS) [20,77]. GSH, a non-enzymatic radical scavenger, plays an essential role in maintaining cellular function and preventing oxidative damage in the brain, particularly under conditions of oxidative stress [78,79]. As the most abundant endogenous antioxidant in neurons, dysregulation of GSH has been implicated in various psychiatric disorders [28]. SOD, a key endogenous antioxidant enzyme, mitigates oxidative stress by catalyzing the conversion of superoxide radicals into hydrogen peroxide [78,80]. Decreased SOD activity, which is commonly observed in major depressive disorder (MDD), reflects impaired antioxidant defense; however, antidepressant treatments have been shown to restore its activity [81]. Consistent with those of previous studies, our findings indicate that chronic stress exposure reduces both SOD and GSH activities, suggesting compromised antioxidant defense and disrupted cellular redox balance. Notably, agomelatine treatment alleviates these impairments. Previous studies suggest that chronic social defeat stress is linked to disruptions in glutamine–glutamate cycling in astrocytes, implying that GSH depletion under such stress may result from both oxidative stress effects and increased demand for glutamate precursors [28,82]. Furthermore, LDH, an oxidoreductase involved in carbohydrate glycolysis [83], serves as a marker for oxidative stress [79]. In our previous research, reduced LDH levels were observed in the CA1, DG, and vmPFC regions of chronic unpredictable stress (CUS)-exposed rats [77]. ROS, the most important oxidants, are endogenous byproducts of oxidative respiration, intracellular signaling molecules, and defense substances [84]. The equilibrium between reactive oxygen species (ROS) production and scavenging is critical in determining the extent of oxidative stress [76]. Under physiological conditions, ROS are generated at controlled levels and neutralized by antioxidants to preserve cellular homeostasis. However, when ROS generation surpasses the capacity of the antioxidant defense mechanisms, oxidative stress ensues, resulting in cellular damage [85,86]. Elevated ROS levels have been implicated in depression, as supported by findings of increased ROS in patient serum samples [15,74]. Dihydroethidium (DHE), a fluorescent probe, is widely utilized for assessing ROS production, particularly superoxide anions [87,88]. In our present study, we observed that chronic social defeat stress (CSDS) exposure led to increased lactate dehydrogenase (LDH) activity and enhanced fluorescence intensity of DHE staining. Notably, agomelatine effectively mitigated these alterations.

The nuclear factor-E2-related factor 2 (Nrf2)/heme oxygenase-1 (HO-1) pathway constitutes a pivotal mechanism for mitigating oxidative stress by modulating the expression of antioxidant genes, including HO-1, superoxide dismutase (SOD), and NAD(P)H:quinone oxidoreductase 1 (NQO1) [89,90,91]. Under conditions of elevated reactive oxygen species (ROS), Nrf2 translocates to the nucleus, where it binds to antioxidant response elements (AREs) within the promoter regions of target genes, thereby activating their transcription to maintain redox homeostasis and protect cells from oxidative damage [92]. Notably, HO-1, a key downstream effector of Nrf2, plays a critical role in antioxidant defense by catalyzing the breakdown of heme into biliverdin, iron, and carbon monoxide, which collectively contribute to reducing oxidative damage [93]. Emerging evidence underscores the significant association between the Nrf2/HO-1 pathway and major depressive disorder (MDD) [94]. Furthermore, agomelatine has demonstrated antioxidant properties via the Nrf2/HO-1 pathway in various pathological conditions, such as acute pancreatitis, cerebral ischemia, lung injury, and neuropathic pain [95,96,97,98]. However, the extent to which the Nrf2/HO-1 pathway plays a role in mediating the antidepressant effects of agomelatine remains to be elucidated. In this study, we observed that chronic social defeat stress (CSDS)-exposed mice exhibited diminished the protein expression levels of Nrf2 and HO-1 in the hippocampus, which were markedly restored following agomelatine treatment. Notably, agomelatine’s activation of the Nrf2/HO-1 pathway aligns with the concept of hormesis, wherein bioactive compounds enhance brain resilience and neuroprotection against stress-related challenges [99,100].

Oxidative stress, arising from an imbalance between reactive oxygen species (ROS) and antioxidants, can induce significant damage to lipids, proteins, and DNA, thereby altering cellular functions [69,101]. Key markers of oxidative damage include malondialdehyde (MDA), 4-hydroxynonenal (4-HNE), and 8-hydroxy-2′-deoxyguanosine (8-OHdG) [102]. MDA, a byproduct of lipid peroxidation, is a well-established marker of lipid damage, with elevated levels signifying extensive lipid membrane impairment [86]. Similarly, 4-HNE, derived from the peroxidation of linolenic and arachidonic acids, is a potent neurotoxic compound [103]. Elevated plasma levels of both MDA and 4-HNE have been observed in patients with depression [20]. Moreover, 8-OHdG, which marks oxidative damage to DNA, reflects alterations in nucleic acids and is also elevated in depressed individuals [104,105]. In our study, chronic social defeat stress (CSDS) significantly increased MDA activity and the fluorescence intensity of 4-HNE and 8-OHdG. Notably, treatment with agomelatine effectively mitigated these oxidative injuries, suggesting its potential protective role. These findings provide compelling evidence that agomelatine may play a crucial role in alleviating depression-like behaviors induced by CSDS exposure, likely through a reduction in oxidative stress. Unlike serotonin–norepinephrine reuptake inhibitors (SNRIs), which exhibit limited effects on oxidative stress [106,107], and selective serotonin reuptake inhibitors (SSRIs), which primarily modulate oxidative stress by enhancing antioxidant enzyme synthesis, inhibiting ROS-generating pathways, and suppressing immune activation [108], agomelatine exerts a broader and more direct antioxidative effect through both MT1/MT2 receptor-dependent and receptor-independent mechanisms, including ROS scavenging, modulation of redox homeostasis, and activation of the Nrf2/HO-1 pathway [71,98]. Notably, melatonin, the endogenous ligand of MT1/MT2 receptors, exerts antioxidative effects via similar pathways, functioning as a ROS scavenger, enhancing antioxidant enzyme activity, and mitigating oxidative damage [109,110]. Given these shared mechanisms, agomelatine’s antioxidative properties are closely linked to its melatonergic activity. It is worth noting that C57BL/6J mice exhibit variability in melatonin biosynthesis, with some studies reporting deficiencies [111,112] and others suggesting retained synthesis capability [113]. However, since agomelatine acts directly on MT1/MT2 receptors, its effects are not necessarily reliant on endogenous melatonin production. Nevertheless, further investigation is warranted to clarify the specific mechanisms by which agomelatine alleviates CSDS-induced oxidative stress.

Mitochondria are both the primary source and target of ROS [114]. Oxidative stress can impair mitochondrial function by disrupting oxidative phosphorylation and interfering with electron transfer in the mitochondrial respiratory chain [89]. Therefore, we hypothesize that the protective effect of agomelatine against oxidative stress in CSDS mice may involve the enhancement of mitochondrial function. To further investigate mitochondrial dysfunction in hippocampal tissue, we performed transmission electron microscopy (TEM). Our findings revealed mitochondrial damage, including mitochondrial swelling and the loss of cristae in the hippocampi of mice exposed to CSDS, which was markedly reversed by intraperitoneal injection of agomelatine. Cytochrome C, a key mitochondrial protein, is released into the cytoplasm when mitochondrial permeability transition pores (mPTPs) open as a result of oxidative stress, disrupting normal cellular function [115]. Elevated levels of cytochrome C have been detected in the hippocampi of chronic unpredictable mild stress (CUMS)-exposed mice [116]. In this study, we observed that CSDS exposure elevated cytochrome C expression in the hippocampus, which was reduced following agomelatine treatment.

Synapses, which have high energy demands due to their critical roles in synaptic transmission and calcium buffering, rely heavily on mitochondria for ATP production [117,118,119,120]. Given their substantial energy requirements and limited glycolytic capacity, synapses are particularly susceptible to oxidative stress, which can impair mitochondrial function and disrupt energy supply, potentially leading to neuronal dysfunction [85]. Consequently, we hypothesize that the antidepressant effects of agomelatine may be associated with its impact on synaptic function. Vesicular glutamate transporter 1 (VGLUT1), a key protein involved in glutamate transport into synaptic vesicles, serves as a marker for glutamatergic synapses [121]. Postsynaptic density protein 95 (PSD95) is a crucial scaffolding protein located at the postsynaptic membrane, where it regulates synaptic transmission and plasticity by organizing receptors and signaling molecules [122,123]. The colocalization of VGLUT1 and PSD95 is commonly used to assess the density and activity of excitatory synapses [124,125]. In our study, we observed a significant reduction in VGLUT1/PSD95 colocalization in chronic social defeat stress (CSDS)-exposed mice. However, treatment with agomelatine mitigated this reduction, suggesting that agomelatine reversed the synaptic alterations induced by CSDS. Furthermore, exposure to CSDS decreased the frequency and amplitude of miniature excitatory postsynaptic currents (mEPSCs) in the hippocampus. Agomelatine injection alleviated these changes, indicating that modifications in synaptic transmission and excitability in pyramidal neurons may contribute to the antidepressant effects of agomelatine. Taken together, these findings suggest that the reversal of synaptic dysfunction could play a role in the antidepressant mechanism of agomelatine.

In conclusion, our study provides compelling evidence that agomelatine effectively mitigates depressive and anxiety-like behaviors induced by chronic social defeat stress. This protective effect is likely mediated through the attenuation of oxidative stress, mitochondrial dysfunction, and synaptic impairment. However, several limitations should be acknowledged. First, as this study relied on an animal model, the findings may not fully translate to human depression. Additionally, while we focused on oxidative stress in the hippocampus, the effects of agomelatine on oxidative stress in other brain regions, as well as its potential interactions with other factors, such as HPA axis dysregulation and genetic influences, were not examined. Notably, the melatonin proficiency status of the C57BL/6J mice was not verified, which may impact stress responses and receptor sensitivity. Furthermore, although agomelatine’s antioxidative effects were demonstrated, the relative contributions of receptor-dependent and receptor-independent mechanisms remain unclear. Given these limitations, future studies should assess melatonin synthesis capability in experimental animals, explore the interplay with stress-related pathways, extend investigations beyond the hippocampus, and employ selective receptor antagonists to further elucidate agomelatine’s mechanisms of action. A deeper understanding of these pathways will be critical for optimizing agomelatine’s therapeutic potential in the treatment of depression.

## 5. Conclusions

In summary, the findings of this study indicate that chronic social defeat stress (CSDS) induces substantial oxidative stress in the hippocampus, thereby contributing to depressive and anxiety-like behaviors in mice. Treatment with agomelatine effectively alleviates these stress-induced behavioral impairments by modulating oxidative stress, restoring mitochondrial function, and preventing synaptic damage. The therapeutic efficacy of agomelatine is associated with activation of the Nrf2/HO-1 pathway, which plays a pivotal role in antioxidant defense mechanisms. Additionally, agomelatine mitigated markers of oxidative damage, including MDA, 4-HNE, and 8-OHdG, and protected against mitochondrial damage, such as mitochondrial swelling and cytochrome C release. Importantly, synaptic function, evaluated through the density of excitatory synapses (VGLUT1/PSD-95 colocalization) and synaptic transmission (mEPSC recordings), was significantly restored following agomelatine treatment, underscoring its neuroprotective role in maintaining synaptic plasticity. Notably, depression is a multifaceted clinical disorder presenting with complex and heterogeneous symptoms, a high degree of resistance to antidepressant therapies, and the potential for adverse reactions that significantly reduce patient compliance with pharmacological treatments. These challenges highlight the necessity of elucidating the mechanisms underlying antidepressant efficacy to enhance therapeutic strategies. Our findings underscore the potential of agomelatine as a promising therapeutic strategy for treating depression and anxiety, particularly by targeting oxidative stress and neuroplasticity mechanisms in the hippocampus. Further research is warranted to elucidate the precise molecular mechanisms underlying the neuroprotective effects of agomelatine in stress-related disorders.

## Data Availability

The data that support the findings of this study are available from the corresponding author upon reasonable request.

## Supplementary Materials

The following supporting information can be downloaded at: www.mdpi.com/article/10.3390/antiox14040410/s1, Table S1: Statistical data of experimental results.

## Abbreviations

The following abbreviations are used in this manuscript:

MDD	major depressive disorder
ROS	reactive oxygen species
MDA	malondialdehyde
GSH	glutathione
SOD	superoxide dismutase
LDH	lactate dehydrogenase
BDNF	brain-derived neurotrophic factor
SSRIs	selective serotonin reuptake inhibitors
SNRIs	serotonin–norepinephrine reuptake inhibitors
TBSIT	three-chambered social interaction test
BSA	bovine serum albumin
TEM	transmission electron microscopy
ACSF	artificial cerebrospinal fluid
8-OHdG	8-hydroxy-2′-deoxyguanosine
Nrf2	nuclear factor erythroid 2-related factor 2
HO-1	heme oxygenase-1
DHE	dihydroethidium
4-HNE	4-hydroxynonenal
Cytc	cytochrome C
VGLUT1	vesicular glutamate transporter 1
PSD-95	postsynaptic density protein-95
BCA	bicinchoninic acid
mEPSCs	miniature excitatory postsynaptic currents
CRS	chronic restraint stress
CMS	chronic mild stress
DAPI	4′, 6-diamidino-2-phenylindole dihydrochloride
CUMS	chronic unpredictable mild stress
TST	tail suspension test
FST	forced swim test
EPM	elevated plus maze
OFT	open field test
SPT	sucrose preference test
i.p.	intraperitoneal
PBS	phosphate buffer saline
PFA	paraformaldehyde.
SIT	social interaction test
PPP	pentose phosphate pathway
CSDS	chronic social defeat stress
ANOVA	analysis of variance
