# Supplementary material for: Agomelatine Alleviates Depressive-like Behaviors by Suppressing Hippocampal Oxidative Stress in the Chronic Social Defeat Stress Model
Source: Antioxidants (Basel). 2025 Mar 28;14(4):410. doi: 10.3390/antiox14040410 (PMC12024063; doi:10.3390/antiox14040410)
Supplement: Supplementary file 1 [file antioxidants-14-00410-s001.zip › antioxidants-3477298-supplementary.pdf]

Supplementary Table S1. Statistical data of experimental results

Figure 1B

SIT-social interaction ratio							Average	SD	SEM
Control	1.0694	1.04718	1.02142	1.38471	1.47612	1.26084	1.21	0.1928	0.07869
CSDS	0.53688	0.21973	0.27182	0.52423	0.74714	0.49063	0.4651	0.1931	0.07883

Figure 1D

FST-climbing time (s)							Average	SD	SEM
Control	149	115	145	125	132	119	130.8	13.83	5.648
CSDS	45	64	65	37	34	28	45.5	15.71	6.412

Figure 1E

FST-immobility time (s)							Average	SD	SEM
Control	24	64	46	14	34	68	41.67	21.67	8.846
CSDS	163	166	134	138	118	73	132	34.16	13.95

Figure 1G

TST-immobility time (s)							Average	SD	SEM
Control	35	60	56	52	49	47	49.83	8.658	3.535
CSDS	97	95	98	106	85	83	94	8.626	3.521

Figure 1I

EPM-Time in open arms (s)							Average	SD	SEM
Control	54.9	185.2	109.5	72.88	78.08	55.84	92.73	49.46	20.19
CSDS	36.48	39.56	31.28	33.2	37.7	33.6	35.3	3.123	1.275

Figure 2B

8-OHdG relative intensity							Average	SD	SEM
Control	1.165	1.06711	1.19147	0.85792	1.09379	0.62471	1	0.2183	0.08911
CSDS	3.20231	1.55476	1.91426	2.74233	2.47453	1.4725	2.227	0.6924	0.2827

Figure 2C

MDA activity (nmol/mg prot)							Average	SD	SEM
Control	3.45559	2.64407	2.76252	2.24347	2.67899	2.8472	2.772	0.3943	0.161
CSDS	9.64254	6.18435	4.09721	6.91631	4.38524	5.86328	6.181	2.01	0.8204

Figure 2E

DHE relative intensity							Average	SD	SEM
Control	0.51138	1.11915	0.81328	1.00853	1.07349	1.47416	1	0.3219	0.1314
CSDS	1.68706	2.81736	2.09367	3.3155	3.74283	3.41465	2.845	0.8072	0.3295

Figure 2F

SOD activity (U/mg prot)							Average	SD	SEM
Control	47.3842	33.0131	50.6001	46.7559	74.3455	64.929	52.84	14.65	5.982
CSDS	13.5062	11.0837	4.8474	34.4929	18.9394	26.2548	18.19	10.79	4.404

Figure 2H

Nrf2 protein level							Average	SD	SEM
Control	0.6233	0.47737	0.63207	0.7861	0.73673	0.38559	0.6069	0.1521	0.06208
CSDS	0.42165	0.45893	0.2984	0.54324	0.35112	0.33652	0.4016	0.09076	0.03705

Figure 2I

HO-1 protein level							Average	SD	SEM
Control	1.40125	0.88295	0.92453	1.23339	0.89305	1.31006	1.401	0.5183	0.0954
CSDS	0.89503	0.38116	0.6787	0.86858	0.48124	0.88641	0.895	0.5139	0.0915

Figure 3C

SIT-social interaction ratio									Average	SD	SEM
Control	1.62631	2.2141	1.12798	2.17123	2.14711	1.36849	1.11135	1.54455	1.664	0.4612	0.1631
CSDS	0.21147	0.24198	0.20882	0.11283	0.15385	0.42059	0.01413	0.02012	0.173	0.1317	0.04656
CSDS+Vehicle	0.33094	0.53148	0.27155	0.0797	0.54475	0.46154	0.0708	0.1057	0.2996	0.2001	0.07073
CSDS+AGO	1.03904	1.14125	0.89696	0.50728	1.24644	0.43424	0.41523	0.30984	0.7488	0.3721	0.1315

Figure 3E

TBSIT-Stage1-interaction time (s)									Average	SD	SEM
Control empty	58.6	47.6	59.4	89.6	83.6	44.8	63.4	63	63.75	15.71	5.555
Control stranger1	149	323.9	127	169.8	185.9	211.2	308.1	280.4	219.4	75.26	26.61
CSDS empty	123.4	73	144.5	291.1	143.6	220.5	203.6	104.5	163	70.89	25.06
CSDS stranger1	34.2	101.4	263.5	115.1	73.1	128.3	172	166	131.7	70.02	24.75
CSDS+Vehicle empty	109.8	99.4	60.6	85.1	133.2	126.8	81.5	117.7	101.8	24.85	8.785
CSDS+Vehicle stranger1	111.4	128.6	73.4	129.2	27.8	232.5	144.9	178.9	128.3	62.21	22
CSDS+AGO empty	100.6	63	42	53	22.6	97.6	129.2	67.4	71.93	34.95	12.36

CSDS+AGO stranger1	253.6	316.4	346.9	210.6	186.2	161.2	159.2	298.9	241.6	73.09	25.84

Figure 3F

TBSIT-Stage1-preference index									Average	SD	SEM
Control	2.54266	6.80462	2.13805	1.89509	2.22368	4.71429	4.85962	4.45079	3.704	1.764	0.6238
CSDS	0.27715	1.38904	1.82353	0.397	0.50905	0.58186	0.84479	1.58852	0.9264	0.5926	0.2095
CSDS+Vehicle	1.01457	1.29376	1.21122	1.51821	0.20871	1.8336	1.77791	1.51997	1.297	0.5195	0.1837
CSDS+AGO	2.52087	5.02222	8.25952	3.97358	8.23894	1.65164	1.2322	4.43472	4.417	2.708	0.9573

Figure 3G

TBSIT-Stage2-interaction time (s)									Average	SD	SEM
Control stranger1	66.1	93.2	91.9	52.2	70.6	71.8	93.9	48.8	73.56	18.02	6.37
Control stranger2	128	140.9	122.6	178.8	180.7	170.2	155.9	138.6	152	22.78	8.053
CSDS stranger1	22.4	64.7	188.3	149.4	159.4	147.4	268.8	277.5	159.7	88.42	31.26
CSDS stranger2	52.8	82.6	255.6	212.7	188.9	190.8	76	61.4	140.1	79.99	28.28
CSDS+Vehicle stranger1	40.4	78.4	60.7	227.1	187.8	277.8	111.5	228.3	151.5	89.77	31.74
CSDS+Vehicle stranger2	166.1	156.8	228.8	66.8	213.2	66.4	79.2	181.9	144.9	65.73	23.24
CSDS+AGO stranger1	45.8	51.8	66.9	24.4	126.7	74.6	37.4	191.4	77.38	55.56	19.64
CSDS+AGO stranger2	354.9	164.2	306.1	240.4	140.8	197.6	265.5	192.2	232.7	73.16	25.87

Figure 3H

TBSIT-Stage2-preference index									Average	SD	SEM
Control	1.93646	1.5118	1.33406	3.42529	2.55949	2.37047	1.66028	2.84016	2.205	0.7224	0.2554
CSDS	2.35714	1.27666	1.35741	1.4237	1.18507	1.29444	0.28274	0.22126	1.175	0.6786	0.2399
CSDS+Vehicle	0.75302	2	1.523	0.29414	1.13525	0.23902	0.71031	0.79676	0.9314	0.5997	0.212
CSDS+AGO	7.74891	3.16988	4.57549	9.85246	1.11129	2.64879	7.09893	1.00418	4.651	3.266	1.155

Figure 4B

SPT-sucrose preference(%)									Average	SD	SEM
Control	90	92.8571	98.0392	92.3077	85.9375	91.6667	89.6552	95.4546	91.99	3.696	1.307
CSDS	82.2785	86.1111	77.5862	84.507	87.1429	78.6517	71.4286	79.3103	80.88	5.2	1.839
CSDS+Vehicle	52.381	52.381	76.4706	72.7273	70.4545	49.2308	86.2745	84.7222	68.08	14.9	5.268
CSDS+AGO	80.3922	89.1892	89.3939	84.2105	88.8889	90.3846	86.1538	90.1961	87.35	3.524	1.246

Figure 4C

TST-immobility time (s)									Average	SD	SEM
Control	4	32	2	37	20	38	37	14	23	15.07	5.328
CSDS	89	120	127	99	146	115	121	69	110.8	24.11	8.525
CSDS+Vehicle	126	116	95	137	147	118	102	94	116.9	19.35	6.841
CSDS+AGO	52	10	42	21	52	41	25	22	33.13	15.7	5.55

Figure 4D

FST-immobility time (s)									Average	SD	SEM
Control	62	26	68	9	29	53	56	27	41.25	21.14	7.473
CSDS	146	138	86	156	96	81	153	103	119.9	31.46	11.12
CSDS+Vehicle	99	154	127	130	122	89	119	146	123.3	21.72	7.681
CSDS+AGO	76	40	56	25	27	62	53	40	47.38	17.58	6.216

Figure 4F

EPM-time in open arms (s)									Average	SD	SEM
Control	61.8	43.84	45.32	62.08	56.32	27.28	57.6	79.8	54.26	15.59	5.513
CSDS	36.12	25.08	7.2	9.96	22.52	12.28	25.6	40.2	22.37	12.02	4.248
CSDS+Vehicle	12.68	13.12	19.48	12.2	16.4	19.2	10.4	25.8	16.16	5.119	1.81
CSDS+AGO	29.88	34.08	38.52	16.44	37.04	57.1	44.6	49	38.33	12.4	4.383

Figure 4I

OFT-center time (s)									Average	SD	SEM
Control	25	22.2	27.2	29.6	41.4	31.8	33.23	20.75	28.9	6.69	2.365
CSDS	10.4	10	8	10.4	4.8	3.04	8.12	0.88	6.955	3.632	1.284
CSDS+Vehicle	6.8	14.96	3.44	8.48	13.2	2.4	11.2	13	9.185	4.68	1.655
CSDS+AGO	15.2	29.1	16.2	20.15	20.92	17.92	19.44	26.6	20.69	4.863	1.719

Figure 4J

OFT-distance (m)									Average	SD	SEM
Control	11.9848	12.5149	12.9222	13.7032	12.2276	9.27303	9.50759	11.3559	11.69	1.574	0.5565
CSDS	11.2817	11.9665	12.5768	10.3506	9.81646	9.51982	9.15894	13.7973	11.06	1.634	0.5779
CSDS+Vehicle	11.0909	9.46636	9.9573	12.3881	10.786	13.4873	10.4588	12.2683	11.24	1.366	0.4828
CSDS+AGO	13.0474	12.601	10.1051	10.6629	12.7665	11.8273	10.7506	9.63197	11.42	1.308	0.4623

Figure 5A

MDA activity (nmol/mg prot)							Average	SD	SEM
Control	8.29608	7.14203	8.16485	12.61	8.70135	5.81563	8.455	2.286	0.9332
CSDS	13.72	14.9716	16.179	17.5022	11.9405	13.8162	14.69	1.973	0.8056
CSDS+Vehicle	16.1494	9.94697	13.0663	8.29447	12.0328	10.5203	11.67	2.751	1.123
CSDS+AGO	8.08158	6.2815	7.49678	5.07633	4.66937	4.32729	5.989	1.554	0.6344

Figure 5B

SOD activity (U/mg prot)							Average	SD	SEM
Control	146.398	132.842	154.419	200.748	212.271	98.3605	157.5	42.68	17.42
CSDS	64.9871	121.483	19.8422	136.993	38.3166	97.6443	79.88	46.61	19.03
CSDS+Vehicle	78.4754	71.846	72.9889	51.8658	99.5118	70.714	74.23	15.34	6.263
CSDS+AGO	183.578	217.265	239.041	210.697	277.263	127.909	209.3	50.7	20.7

Figure 5C

GSH activity (μg/mg)							Average	SD	SEM
Control	20.2668	16.9886	13.1475	11.3465	9.72009	23.7649	15.87	5.452	2.226
CSDS	5.38709	4.28234	7.10662	6.81927	8.62293	6.36224	6.43	1.492	0.6089
CSDS+Vehicle	14.1801	9.96281	2.28875	11.1935	8.61032	7.48028	8.953	4.002	1.634
CSDS+AGO	21.8514	31.5718	22.2414	13.1108	17.1913	26.7454	22.12	6.577	2.685

Figure 5D

LDH activity (U/mg prot)							Average	SD	SEM
Control	570.968	581.887	489.628	689.123	688.73	553.234	595.6	79.04	32.27
CSDS	1127.71	965.613	1167.45	932.154	1483.06	1916.9	1265	374.5	152.9
CSDS+Vehicle	1054.57	858.597	722.046	1129.54	1453.04	1409.93	1105	291.4	119
CSDS+AGO	792.081	344.633	428.249	458.039	522.103	432.441	496.3	155.8	63.59

Figure 5F

Nrf2 protein level							Average	SD	SEM
Control	0.68505	0.7909	0.84308	0.691	0.65824	0.66875	0.7228	0.07567	0.03089
CSDS	0.49947	0.67753	0.58339	0.59899	0.51364	0.52263	0.5659	0.06764	0.02762
CSDS+Vehicle	0.52036	0.67074	0.55394	0.57156	0.60129	0.36128	0.5465	0.104	0.04247
CSDS+AGO	0.72559	0.97383	0.71048	0.84651	0.56348	0.75795	0.763	0.1381	0.0564

Figure 5G

HO-1 protein level							Average	SD	SEM
Control	1.07323	0.76769	0.60533	0.88942	0.87901	0.88064	0.8492	0.1548	0.06319
CSDS	0.65944	0.55302	0.75872	0.59479	0.79643	0.8658	0.7047	0.122	0.04981
CSDS+Vehicle	0.60533	0.52454	0.80142	0.56373	0.5822	0.662	0.6232	0.09853	0.04022
CSDS+AGO	0.88942	0.69971	0.8579	0.85677	0.9143	0.71061	0.8215	0.09265	0.03783

Figure 5H

Cytc protein level							Average	SD	SEM
Control	0.37366	0.4678	0.48965	0.54647	0.75578	0.64372	0.5462	0.1361	0.05554
CSDS	0.72403	0.92268	0.52367	0.80433	0.79035	0.92318	0.7814	0.1486	0.06066
CSDS+Vehicle	0.8694	0.78991	0.77912	0.92971	0.80738	0.83391	0.8349	0.0567	0.02315
CSDS+AGO	0.63812	0.69746	0.63156	0.59725	0.73915	0.75882	0.6771	0.06467	0.0264

Figure 5J

DHE relative intensity							Average	SD	SEM
Control	0.9494	1.4792	0.98231	1.07725	0.9701	0.54174	1	0.2998	0.1224
CSDS	2.62305	2.36048	2.34837	2.21644	2.13673	2.6461	2.389	0.2082	0.085
CSDS+Vehicle	2.46864	2.37709	1.57553	2.40183	2.91891	2.77941	2.42	0.4681	0.1911
CSDS+AGO	1.36793	1.24896	1.82483	1.80502	1.9473	1.93214	1.688	0.3015	0.1231

Figure 6B

4-HNE relative intensity							Average	SD	SEM
Control	1.49461	1.23268	1.42793	0.71603	0.45435	0.6744	1	0.4396	0.1795
CSDS	3.66058	3.47639	4.77137	3.24945	4.39444	3.15815	3.785	0.6534	0.2668
CSDS+Vehicle	3.06332	2.97573	5.12356	2.18674	3.82973	2.62823	3.301	1.045	0.4265
CSDS+AGO	0.47323	0.14353	1.18385	0.27671	1.27092	2.21789	0.9277	0.7865	0.3211

Figure 6D

8-OHdG relative intensity							Average	SD	SEM
Control	0.90951	0.88685	0.89686	1.37344	0.83681	1.09654	1	0.2035	0.08307
CSDS	3.60387	2.69815	3.73648	2.06077	2.73384	1.90458	2.79	0.7597	0.3101
CSDS+Vehicle	3.816	3.97966	2.06698	2.67719	2.62999	2.45475	2.937	0.776	0.3168
CSDS+AGO	1.18191	1.22687	0.86001	0.83933	0.68453	0.6902	0.9138	0.237	0.09676

Figure 7B

VGLUT1/PSD-95 synapses (number/mm2)							Average	SD	SEM
Control	10471.3	5890.13	6871.81	5235.67	4253.98	5562.9	6381	2178	889.2
CSDS	2290.6	1963.38	2290.6	1308.92	3926.75	2617.83	2400	869.9	355.1
CSDS+Vehicle	2617.83	1636.15	1963.38	2945.06	1308.92	3926.75	2400	963.3	393.3
CSDS+AGO	3272.29	4253.98	5235.67	2290.6	5562.9	5235.67	4309	1299	530.5

Figure 7D

mEPSC amplitude (pA)							Average	SD	SEM
Control	14.9973	12.4359	18.0816	19.4805	21.1009	20.6647	17.79	3.424	1.398
CSDS	6.4085	7.019	6.104	6.8665	6.86675	6.307	6.595	0.3704	0.1512
CSDS+Vehicle	9.15533	11.902	11.7188	11.2915	10.4982	10.8338	10.9	1.004	0.4098
CSDS+AGO	18.572	22.7865	17.7	19.1826	18.9888	19.455	19.45	1.746	0.713

Figure 7E

mEPSC frequency (Hz)							Average	SD	SEM
Control	2.27879	2.60434	2.60434	2.27879	2.60434	2.27879	2.442	0.1783	0.07279
CSDS	0.65108	1.30217	0.32554	1.30217	1.30217	0.97663	0.9766	0.4118	0.1681
CSDS+Vehicle	2.27879	2.27879	1.95325	1.30217	1.30217	1.62771	1.79	0.4487	0.1832
CSDS+AGO	3.58096	2.27879	3.9065	2.92988	2.60434	3.25542	3.093	0.609	0.2486
